# Nanosatellites for Biology in Space: In Situ Measurement of *Bacillus subtilis* Spore Germination and Growth after 6 Months in Low Earth Orbit on the *O/OREOS* Mission

**DOI:** 10.3390/life10010001

**Published:** 2019-12-29

**Authors:** Wayne L. Nicholson, Antonio J. Ricco

**Affiliations:** 1 Department of Microbiology and Cell Science, University of Florida, Merritt Island, FL 32953, USA; 2 NASA Ames Research Center, Moffett Field, CA 94035, USA; tony.ricco@nasa.gov

**Keywords:** astrobiology, *Bacillus subtilis*, cubesat, germination, Low Earth Orbit, microfluidics, nanosatellite, O/OREOS, SESLO, spores

## Abstract

We report here complete 6-month results from the orbiting Space Environment Survivability of Living Organisms (SESLO) experiment. The world’s first and only long-duration live-biology cubesat experiment, SESLO was executed by one of two 10-cm cube-format payloads aboard the 5.5-kg *O/OREOS* (Organism/Organic Exposure to Orbital Stresses) free-flying nanosatellite, which launched to a 72°-inclination, 650-km Earth orbit in 2010. The SESLO experiment measured the long-term survival, germination, metabolic, and growth responses of *Bacillus subtilis* spores exposed to microgravity and ionizing radiation including heavy-ion bombardment. A pair of radiation dosimeters (RadFETs, i.e., radiation-sensitive field-effect transistors) within the SESLO payload provided an in-situ dose rate estimate of 6–7.6 mGy/day throughout the mission. Microwells containing samples of dried spores of a wild-type *B. subtilis* strain and a radiation-sensitive mutant deficient in Non-Homologoous End Joining (NHEJ) were rehydrated after 14, 91, and 181 days in space with nutrient medium containing with the redox dye alamarBlue (aB), which changes color upon reaction with cellular metabolites. Three-color transmitted light intensity measurements of all microwells were telemetered to Earth within days of each 24-hour growth experiment. At 14 and 91 days, spaceflight samples germinated, grew, and metabolized significantly more slowly than matching ground-control samples, as measured both by aB reduction and optical density changes; these rate differences notwithstanding, the final optical density attained was the same in both flight and ground samples. After 181 days in space, spore germination and growth appeared hindered and abnormal. We attribute the differences not to an effect of the space environment per se, as both spaceflight and ground-control samples exhibited the same behavior, but to a pair of ~15-day thermal excursions, after the 91-day measurement and before the 181-day experiment, that peaked above 46 °C in the SESLO payload. Because the payload hardware operated nominally at 181 days, the growth issues point to heat damage, most likely to component(s) of the growth medium (RPMI 1640 containing aB) or to biocompatibility issues caused by heat-accelerated outgassing or leaching of harmful compounds from components of the SESLO hardware and electronics.

## 1. Introduction

Long-term habitation of the spaceflight environment affects the metabolism and physiology of living organisms, primarily due to chronic exposure to reduced (micro-)gravity and increased ionizing radiation from solar and galactic sources. Extensive investigations conducted in spaceflight on “higher” organisms (i.e., macroscopic, multicellular eukaryotes) have resulted in a relative wealth of knowledge regarding microgravity and radiation effects ranging from the whole body to the organ system to the cellular and molecular level in humans [1], animals [2], and plants [3]. However, it has proven more difficult to understand how microscopic single-celled organisms respond to spaceflight stress. From a theoretical perspective, exposure to microgravity results in a number of alterations in a microbial cell’s immediate surroundings, such as loss of convective mass and heat transfer, reduction in mechanical shear forces associated with gravitational sedimentation, and changes in the behavior of liquids at interfaces with gases or solids. Changes in these fundamental physical parameters are thought to alter the rates at which gases, nutrients, signaling molecules, and waste products are exchanged between microbes and their surroundings. Numerous studies have sought to understand how microbes sense and respond to the spaceflight environment (reviewed in [4,5,6,7,8]), but to date no coherent model has been forthcoming.

Historically, most microbiological studies in the spaceflight environment have been performed by astronauts working inside and outside human-tended spacecraft and habitats operating in Low-Earth Orbit (LEO). The number and complexity of such studies have been constrained by limitations of budget, up-mass, astronaut training and expertise, and available crew time. In response to these issues, there has been considerable effort expended over the past two decades in the development and deployment of self-contained autonomous vehicles capable of performing microbiological experiments in space. Advances in the miniaturization of spacecraft control systems, optics, and microfluidics have led to the development of nanosatellites (nanosats), defined as satellites with masses in the 1–15 kg range. Nanosats utilize a standardized modular architecture based on multiples of 10-cm cubes. Each cube is called a unit (U), and nanosats are most often built in 1U, 3U, and 6U configurations [9]. Because of their standardized design, small size and mass, and heavy use of commercial off-the-shelf (COTS) components, nanosats can be built and launched inexpensively as secondary payloads on rockets carrying larger primary payloads. 

In an effort to understand the response of microbes to the spaceflight environment, a number of microbiological experiments have been launched on nanosats. The first was *GeneSat-1*, a 3U nanosat launched from NASA Wallops Flight Facility on an Air Force Minotaur I rocket as a secondary payload on 16 December 2006. *GeneSat-1* was placed in a circular 460-km, 40.5° inclination orbit and served as a demonstration of the technology for cultivating and measuring the growth of *Escherichia coli* cells via optical density and fluorescent detection of green fluorescent protein (GFP) and telemetering the growth data from space to ground receivers [10]. Evaluation of optical density (turbidity) vs. time for two strains of *E. coli* (DH5α carrying plasmid AcGFP and MM294 carrying plasmid p-GREEN; both strains express GFP constitutively) revealed a doubling time during the exponential growth phase of ~50 min in space microgravity and ~35 min for the ground control.

Building on the design of *GeneSat-1*, a second 3U nanosat mission called *PharmaSat* was launched as a secondary payload from NASA’s Wallops Flight Facility on a Minotaur I rocket on 19 May 2009 and was placed into a 459-km, 40.4°-inclination orbit [11]. The objectives of the *PharmaSat* mission were to cultivate the yeast *Saccharomyces cerevisiae* in space and to compare its level of resistance to the antifungal agent voriconazole in space vs. matched ground control samples [11,12]. Comparison of the optically measured results for the zero-voriconazole-dose microwells in microgravity with those on the ground revealed a longer “lag time” for the spaceflight yeast than the ground specimens, whether measured by cell density (turbidimetry) or metabolism (alamarBlue reduction). As for *GeneSat-1*, spaceflight yeast cultures also exhibited a longer doubling time (2.7 h) than those on the ground (1.7 h) during exponential growth as measured by optical density, with comparable differences for alamarBlue reduction. At low and medium voriconazole concentrations, after accounting for the growth-rate difference of the zero-dose controls, there was no difference between spaceflight and ground specimens in either growth rate or metabolic activity.

Building upon the designs of *GeneSat-1* and *PharmaSat*, a third 3U nanosat called *O/OREOS* (Organism/Organic Exposure to Orbital Stresses) was launched on 19 November 2010 as a secondary payload on a Minotaur IV rocket from Kodiak Launch Complex, Alaska, into a 650-km altitude, 72°-inclination orbit. This orbit caused the nanosat to traverse comparatively weak regions of Earth’s magnetic field and dense regions of the inner van Allen Belt as it traveled just beyond the latitudes of the Arctic and Antarctic Circles on each orbit, in addition to sending it through the South Atlantic Anomaly on ~ 30% of its orbits, thus exposing the nanosat’s contents to some 15 times the typical ionizing radiation dose rate received within satellites in lower-inclination orbits comparable to that of the International Space Station (ISS) (52°) [13]. The *O/OREOS* nanosat was unique in that it carried two separate experiments, called SEVO (Space Environment Viability of Organics) and SESLO (Space Environment Survivability of Living Organisms) [14]. The SESLO experiment measured the germination, growth, and metabolism of *Bacillus subtilis* spores after long-term stasis (14, 91, and 181 days) in the space environment.

On 18 April 2014, a fourth 3U nanosat called *SporeSat-1* was launched from Cape Canaveral on a SpaceX Falcon 9 rocket as a secondary payload on the ISS resupply mission CRS-3. *SporeSat-1* was deployed en route to the ISS at an altitude of 325 km and an inclination of 51.6°; it re-entered Earth’s atmosphere 47 days later. The mission’s science objective was to measure germination of fern spores (*Ceratopteris richardii*) as a function of gravity level using differential pairs of calcium ion-sensitive electrodes [15,16]. The experiment was initiated by increasing temperature to 29 °C and establishing artificial gravity via its two 50-mm microcentrifuges. Germination was to have been initiated using red illumination, but the light source failed (a similar failure occurred on the ground control). Nonetheless, the rotating spore-containing disks were held at the desired temperature and rotated at the pre-defined rates (0.06–1 x***g***); differential calcium ion signals were measured from each of the 96 spores aboard the payload, albeit at the background levels (tens of microvolts) expected in absence of germination.

The first 6U nanosat devoted to space microbiology, called *E. coli* Anti-Microbial Satellite (*EcAMSat*), was launched on 12 November 2017 as a secondary payload aboard an Antares rocket from Wallops Flight Facility bound to the ISS, and was deployed from the ISS on 20 November 2017 into a ~390 km, 51.6° inclination orbit. The objective of the *EcAMSat* mission was to measure growth and resistance to the antibiotic gentamicin in comparison to ground controls using two *Escherichia coli* strains, a wild-type and a mutant defective in the stationary-phase and stress-related sigma factor σ^S^ [17]. Results from the experiment indicated that both strains grew and metabolized more slowly in space than the ground control, and that both strains exhibited more susceptibility to gentamicin in space compared to ground controls [18].

The SESLO experiment on *O/OREOS* was unique among the nanosat missions performed to date, in that *B. subtilis* spores were assayed for survival and growth after 91 and 181 days of exposure to spaceflight, far longer than other microbiological nanosat missions. The SESLO science results after 14 and 91 days in space were reported previously [13]. In this communication, we present the data from the complete 181-day experiment and discuss their implications for long-duration microbiological spaceflights.

## 2. Materials and Methods

The heritage and technical details of the *O/OREOS* nanosat and SESLO mission hardware have been described in detail previously [13]. 

The two congenic strains of *B. subtilis* used were wild-type strain 168 (*trpC2*) and mutant strain WN1087 [*trpC2, Δ(ykoV-ligD)::erm*]. Strain WN1087 is deficient in the Non-Homologous End Joining (NHEJ) DNA-repair system responsible for repair of double-strand breaks, causing it to be more sensitive to ionizing radiation than the wild-type strain [13,19]. The SESLO experiment consisted of three “bioblock” modules, each containing 12 sample wells of 75 μL volume each. In both Bioblocks 1 and 2, six wells were loaded with spores of strain 168 and six with strain WN1087. Bioblock 3 carried only 3 wells of each strain; the other six wells were devoted to a separate experiment not reported here. Growth was initiated by filling the wells with filter-sterilized (0.22 μm filter) growth medium consisting of RPMI-1640 Modified, with L-glutamine, without phenol red and sodium bicarbonate (Sigma-Aldrich Cat. No. R8755), buffered with 0.1 M 3-(*N*-morpholino) propanesulfonic acid (MOPS, pH 7.0) and containing the redox dye alamarBlue (aB, the chemical name of which is resazurin) (Invitrogen, Carlsbad, CA, USA) added at a 1:25 dilution of the stock solution [13]. While not a standard *B. subtilis* growth medium, the RPMI 1640 formulation described above had previously been tested for stability and biocompatibility and was used successfully to cultivate *S. cerevisiae* in the prior *PharmaSat* experiment [11,12]. Pre-flight testing of the medium showed it also supported *B. subtilis* growth well, so it was used for the SESLO experiment.

Commands to initiate spore germination and growth were sent to Bioblocks 1, 2, and 3 at 14, 91, and 181 days post-launch, respectively. The resulting data were stored in the onboard memory for later downlink to the receiving station. An asynchronous ground control experiment was performed using identical flight-spare hardware, loaded in parallel to the flight hardware with the same batches of medium and spores. Ground control experiments were performed approximately 6 weeks after flight experiments, which provided time for the external temperature of the SESLO ground system to be approximately matched on a delayed basis to the time-varying temperature telemetered from the thermal sensors of the flight system [13]. To prevent cell sedimentation during the experimental period, the ground-control hardware was mounted on a “rotisserie” apparatus which caused the payload hardware to be rotated back and forth through 355° at a speed of 1.5 rpm.

Bioblock 1 was activated 14 days post-launch on 3 December 2010. First, optical readings were initiated; detector frequencies (proportional to transmitted light intensities) were recorded at the three LED wavelengths (615, 525, and 470 nm) from the 12 microwells of the active bioblock at 2-min intervals throughout the experimental period. Next, the bioblock temperature was adjusted to the growth target, 37 °C, and allowed to stabilize for ~1 h before growth medium was pumped through the microchannels and into the wells, displacing the air through hydrophobic membranes. On 18 February 2011 and 19 May 2011 (91 and 181 days post-launch, respectively), the same procedure was repeated for Bioblocks 2 and 3. Data were stored in the onboard memory for subsequent downlink. In a prior publication [13], preliminary raw data was simply normalized to zero time and reported as ratios. For the present communication, all detector frequencies from all three timepoints were converted to Absorbance values corrected for crosstalk among the three detectors as described in Appendix A. The complete data from the SESLO experiment have been deposited in the NASA Life Science Data Archive (LSDA) as described in the section Supplementary Materials.

## 3. Results

### 3.1. SESLO Experiment at 14 Days

**Figure 1:**
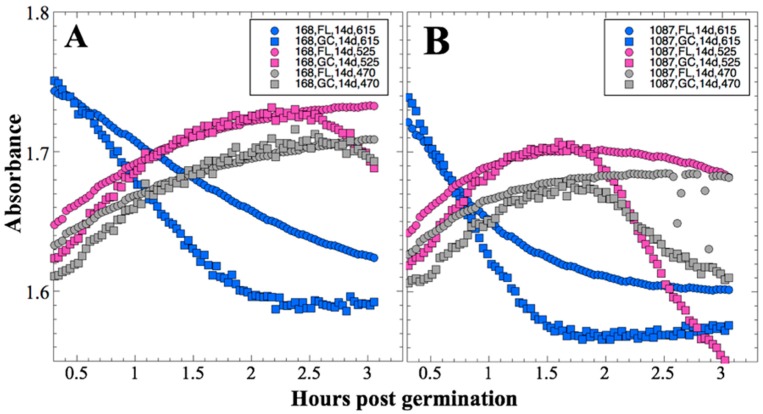
Space Environment Survivability of Living Organisms (SESLO) results 14 days post-launch. Germination and metabolism of *Bacillus subtilis* strain 168 (**A**) and WN1087 (**B**). Flight (FL) and Ground Control (GC) samples are denoted by circles and squares, respectively. Symbol colors: blue symbols denote blue (oxidized) form of aB, measured at 615 nm; magenta symbols denote pink (reduced) form of aB, measured at 525 nm; gray symbols denote (mostly) optical density, measured at 470 nm. Results shown are the averages of six separate wells for each strain. Error bars have been omitted for clarity but are presented as standard deviations of the mean in the datasets deposited in the NASA Life Science Data Archive (LSDA).

Because spore germination and growth, as well as conversion of aB to its pink form, were essentially concluded within the first 3 hours after addition of growth medium, these data are presented in Figure 1 for both flight (FL) and ground-control (GC) experiments. From visual inspection of the data, it appeared that for all three wavelengths the initial rate of metabolism and growth were slower in the FL samples than in the GC samples (Figure 1). Primary reduction of aB, or resazurin, (blue) to resorufin (pink) is irreversible; however, resorufin can further be reversibly reduced to hydroresorufin (colorless), leading to loss of absorbance at 525 nm [20]. This secondary reduction process was also observed to proceed more slowly in the FL than in the GC samples (Figure 1).

Note that, for a given concentration of aB, the rate of color change depends on the number of cells and their production of several electron-donor molecules associated with metabolism, as well as the rate at which unreacted resazurin is transported to the cells, so a larger cell population, greater metabolic activity, more rapid solution mass transport, or any combination of these three factors, can result in more rapid color change. Further, cellular metabolic rate may in some circumstances depend upon the rate of mass transport of nutrients to the cells, an additional mechanism by which solution transport can impact the rate of color change.

**Figure 2:**
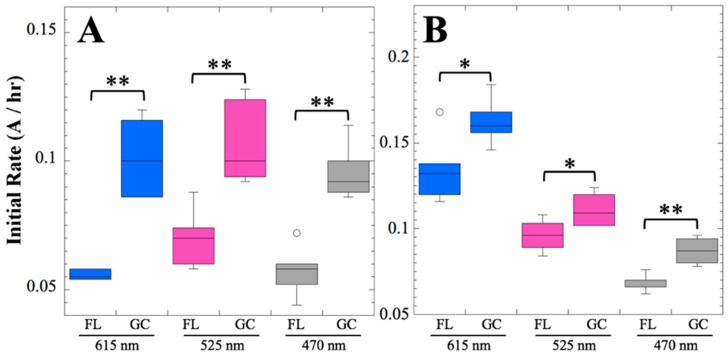
Initial rates of metabolism and growth, 14 days post-launch. The initial rates of absorbance change in strains 168 (**A**) and WN1087 (**B**) FL and GC samples are compared for the oxidized (blue) and reduced (magenta) forms of aB, and optical density (gray). Data are shown as box plots (*n* = 6). Kruskal-Wallis significance is denoted: *, *P* < 0.05; **, *P* < 0.01.

The notion that metabolism and growth and/or mass transport were proceeding more slowly in the FL than in the GC samples at 14 days was tested by measuring the initial slopes of the data at all three wavelengths from each individual well (*n* = 6). Each group of data was then tested for normality using the Shapiro-Wilk method [21]; most, but not all, groups were normally distributed. Because not all groups of data were normally distributed, they were analyzed for differences using a non-parametric statistical method, the Kruskal-Wallis rank test [22]. The initial rate of absorbance change at all three wavelengths was found to be significantly less in FL samples than in the corresponding GC samples, both in strains 168 and WN1087 (Figure 2).

### 3.2. SESLO Experiment at 91 Days

**Figure 3:**
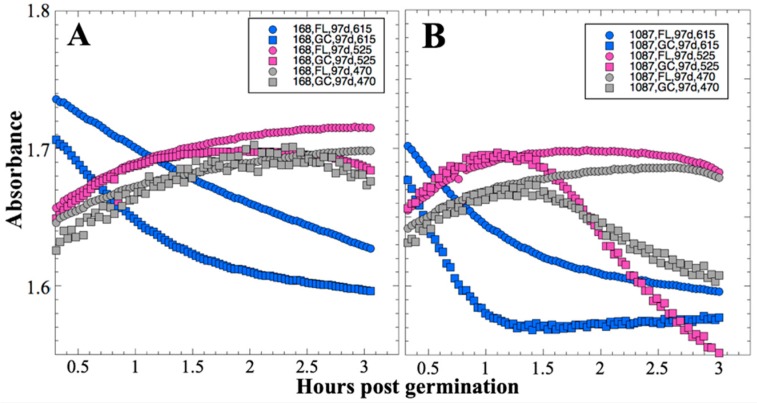
SESLO results 91 days post-launch. Germination and metabolism of *B. subtilis* strain 168 (**A**) and WN1087 (**B**). Flight (FL) and Ground Control (GC) samples are denoted by circles and squares, respectively. Symbol colors: blue symbols denote blue (oxidized) form of aB, measured at 615 nm; magenta symbols denote pink (reduced) form of aB, measured at 525 nm; gray symbols denote (mostly) optical density, measured at 470 nm. Results shown are the averages of six separate wells for each strain. Error bars have been omitted for clarity but are presented as standard deviations of the mean in the data deposited in the LSDA.

The patterns of absorbance change at 91 days were noted to be very similar to those seen at 14 days, in that germination, growth, and conversion of aB to its pink form had mostly concluded by 3 hours after addition of the nutrient medium; these data are presented in Figure 3.

**Figure 4:**
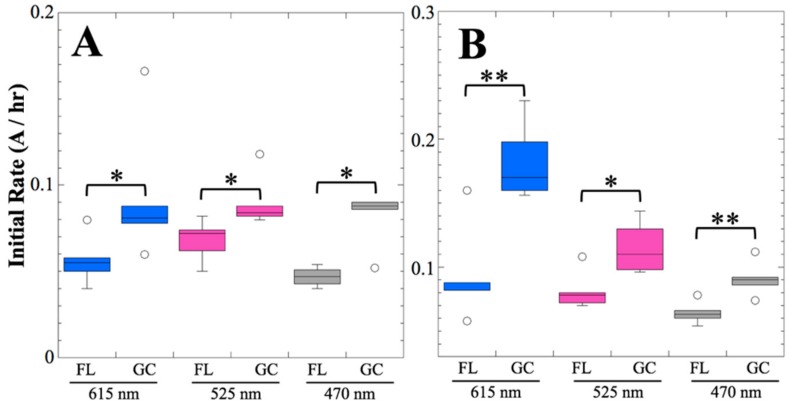
Initial rates of metabolism and growth, 91 days post-launch. The initial rates of absorbance change in strains 168 (**A**) and WN1087 (**B**) FL and GC samples are compared for the oxidized (blue) and reduced (magenta) forms of aB, and optical density (gray). Data are shown as box plots (*n* = 6). Kruskal-Wallis significance is denoted: *, *P* < 0.05; **, *P* < 0.01.

From visual inspection of the data, again it appeared that the FL samples germinated and grew more slowly than the GC samples, or were in a condition of slower aB mass transport, and again it appears that secondary reduction of aB was slower in the FL samples compared to the GC samples (Figure 3). This notion was again tested by measurement of initial slopes of the absorbance data at all three wavelengths for each individual sample well and their comparison using non-parametric statistics. Again, the initial rate of absorbance change at all three wavelengths was found to be significantly less in FL samples than in the corresponding GC samples, both in strains 168 and WN1087 (Figure 4).

### 3.3. Thermal Anomalies at Days 102–116 and Days 162–176

**Figure 5:**
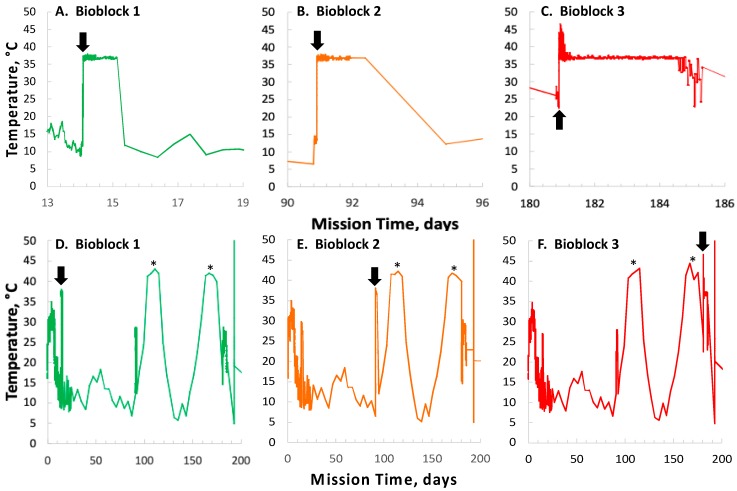
Thermal profiles of SESLO flight. Depicted are thermal profiles of SESLO Bioblocks 1 (**A**,**D**), 2 (**B**,**E**), and 3 (**C**,**F**). Top row (**A**–**C**): thermal stability at 37 °C for each Bioblock around the time of its growth experiment. Bottom row (**D**–**F**): overall thermal profile of each Bioblock over the ~ 6 months in space that included all three SESLO experiments. The two thermal excursions are denoted by asterisks. Vertical arrows on all panels indicate activation times of each Bioblock.

Its high-inclination orbit resulted in the *O/OREOS* nanosat spending variable portions of each trip around the Earth in full sunlight and in Earth’s shadow, thus subjecting it to heating/cooling cycles. Under normal conditions, these thermal cycles were within the capability of the onboard active heating and passive cooling system to maintain the Bioblocks’ temperatures within a nominal range of 4–35 °C when not operating, and at 37 ± 1 °C in operation. The measured thermal performance was (average ± standard deviation): 36.9 ± 0.3 °C, Bioblock 1; 36.9 ± 0.3 °C, Bioblock 2; 36.9 ± 0.2 °C, Bioblock 3. However, at Days 102–116 and again at Days 162–176 post-launch, the nanosat entered an unusual situation in which it spent a very large fraction of each orbit in full sunlight, peaking at nearly 24 hours per day for several days, exceeding the capacity of the thermal control-and-dissipation system to compensate (Figure 5). During this off-nominal condition, onboard sensors recorded temperatures in the three Bioblocks of 41–44 °C for ~12 days each (i.e., 24 days in total), the highest temperatures being recorded in Bioblock 3, which did not feed and grow its microbial cultures until after both thermal anomalies had occurred.

**Figure 6:**
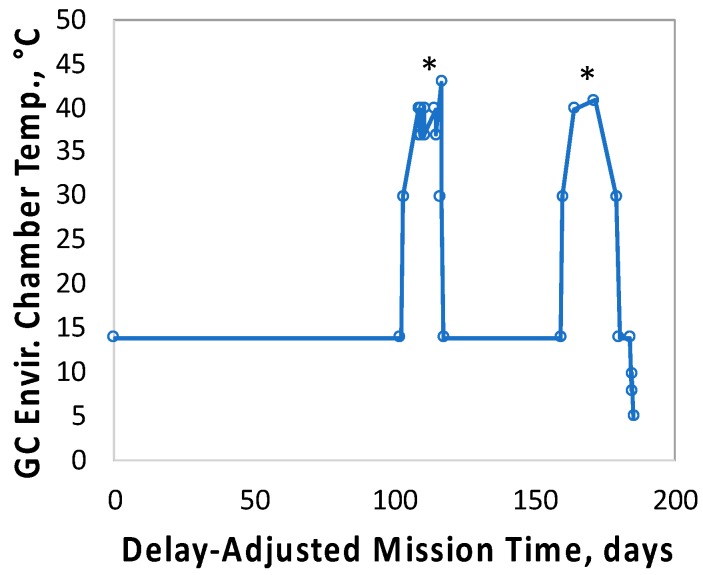
Thermal profile in the environmental chamber containing the SESLO GC unit. The two thermal excursions are denoted by asterisks.

The thermal excursions of the SESLO space payload were matched approximately and asynchronously in the GC SESLO module within a laboratory environmental chamber. The temperature record for the exterior of the SESLO payload, telemetered by the satellite, was used to program the chamber; the programmed temperature sequence, time adjusted for the 50-day delay between FL and GC experiments, is shown in Figure 6.

### 3.4. SESLO Experiment at 181 Days

**Figure 7:**
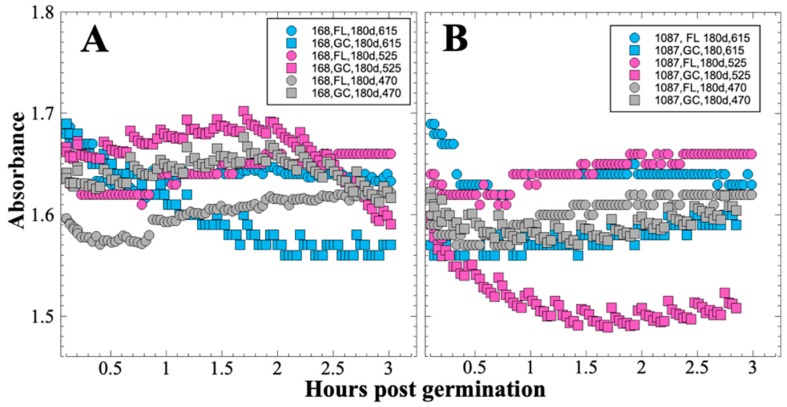
SESLO results 181 days post-launch. Germination and metabolism of *B. subtilis* strain 168 (**A**) and WN1087 (**B**). Flight (FL) and Ground Control (GC) samples are denoted by circles and squares, respectively. Symbol colors: blue symbols denote blue (oxidized) form of aB at 615 nm; magenta symbols denote pink (reduced) form of aB at 525 nm; gray symbols denote (mostly) optical density 470 nm. Results shown are the averages of 3 separate wells for each strain. Error bars have been omitted for clarity but are presented as standard deviations of the mean in data deposited in the LSDA.

Visual inspection of the data revealed that spore germination did not proceed in either the FL or GC samples at 181 days as it had at 14 or at 91 days (Figure 7). Because germination of the spores at 181 days did not proceed normally, we were unable to calculate initial rates of germination for comparison between FL and GC samples.

### 3.5. Ionizing Radiation Dosimetry

**Figure 8:**
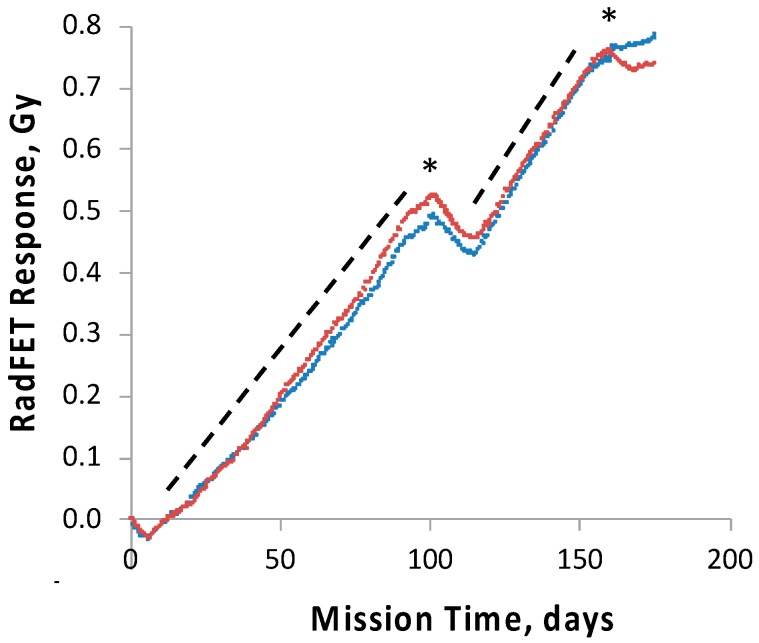
Ionizing radiation dosimetry by the pair of RadFETs (red and blue points) inside the SESLO experiment container. Asterisks correspond to the two thermal excursions experienced by the nanosat (refer to Figure 5 for details). Dashed lines denote regions from which effective TID rates were calculated.

For purposes of measuring the ionizing radiation flux, a pair of radiation-sensitive field-effect transistors (RadFETs) were located within the SESLO payload. This class of device provides a response to ionizing radiation as a consequence of electron–hole pairs formed in the dielectric layer of the RadFET gate [23]; electrons diffuse away, and the positive trapped charge shifts the device threshold voltage in proportion to the total ionizing dose (TID) received. Interpretation and conversion of such threshold voltage shifts to TID is effective when the device operates in a relatively constant-temperature environment. In particular, elevated temperature can “anneal” the damage (effectively untrapping the trapped charge) in the gate dielectric, resulting in the (misleading) appearance of a negative rate of change of TID. Figure 8 shows that such an effect occurred three times during the mission: soon after launch and again during the two thermal anomalies (cf. Figure 5, panels D–F).

Due to *O/OREOS*’s unique orbit and resultant variable thermal conditions across the mission, we estimated TID rates from the SESLO RadFETs only during periods of relative thermal constancy (periods of eclipse during every 98-min orbit, which were comparatively consistent at times apart from the thermal anomalies, do not interfere with such estimation). Linear least-squares fits to the slopes for both RadFETs averaged to 6.0 mGy/day for Mission Day 20–90 and 7.6 mGy/day for Mission Day 118–151; the regions over which these slopes were obtained are shown in Figure 8 by dashed lines. We used the former rate to estimate TIDs of ~0.08 and 0.5 Gy for SESLO at Mission Days 14 and 91, respectively; we used both rates to estimate a TID of ~1.2 Gy at Mission Day 181.

## 4. Discussion

Nanosats for space biology offer a number of advantages over traditional spaceflight experiments involving astronauts, including lower mission costs (assuming the cost of crew and ISS operations are included in such calculations) and fewer demands on crew training and availability. With the exception of the SESLO experiment described here, all live-biology nanosat space experiments conducted to date have been of rather short duration (a week of data collection at most). The shorter durations of most studies were due to two factors. First, the primary goal of the initial studies was to understand microgravity effects [9,10,11,14,15,16], for which there was no reason to delay growth and analysis beyond the time at which spacecraft stabilization occurred (several hours to a few days after deployment). Second, the first two missions, *GeneSat-1* and *PharmaSat*, traversed the steep part of the learning curve for biological nanosat missions—among other things, a very intimate proximity of salty water and electronics—meaning that longevity of the growth-and-measurement system in space for many weeks was far from guaranteed. 

Longer stasis times, conducive to studying the biological effects of prolonged durations in space, are particularly well suited to the use of bacterial spores, which in the dry state have an essentially unlimited shelf life [24]; a months-long science mission was also more appropriate for the third-ever microbiological nanosat, SESLO, than the first two. The results presented above from SESLO represent the outcome of the first nanosat mission testing biological responses of the bacterium *B. subtilis* to long-duration space exposure as well as the first nanosat biology experiment to collect data over a period of months. The mission was designed to test spore germination and growth of *B. subtilis* after exposure to the spaceflight environment for three different times: immediately after deployment, and at approximately 90 and 180 days thereafter (nearly 6 months total). Complete stabilization and establishment of routine command-and-data transfer processes between ground and spacecraft required a number of days, so the “immediate” data point was collected after 14 days in space. 

After 14 and 91 days in space, it was observed that the test organisms, spaceflight hardware, and protocols all performed in a satisfactory manner, such that a high density of reliable data was obtained. Measurements were obtained of (i) metabolic activity (convolved with reaction rate), via the conversion of the oxidized (blue) to the reduced (pink) form of aB at 615 and 525 nm, respectively, and (ii) optical density measured at 470 nm, which mainly results from light scattering by cells.

Several spaceflight experiments have assessed the effects of microgravity on the growth of a variety of bacteria in liquid culture, but no consistent picture has emerged. Depending on the species, strain, hardware, and growth conditions, reports range from spaceflight promoting increased, decreased, or no growth differences between FL and GC samples (reviewed in [4,6,25,26,27]). Due to hardware limitations, most spaceflight experiments only measure the final number of cells in cultures after samples have been returned to Earth. In the SESLO experiment, we measured both growth rates and final optical densities of the cultures at two-minute intervals in situ to obtain kinetic growth data. At both 14 and 91 days, the FL samples were observed to germinate and grow, and/or to react with aB, significantly more slowly than the corresponding GC samples, as measured both by aB reduction and optical density changes (Figure 2 and Figure 4); however, the maximum optical density (bacterial population) achieved was not significantly different between FL and GC samples (Figure 1 and Figure 3). The observations from the SESLO experiment that growth/metabolism was slower in FL compared to GC samples is in good agreement with prior growth/metabolism results obtained by the *GeneSat-1*, *PharmaSat,* and *EcAMSat* experiments.

To track and correlate with observed results the radiation dose received by the science payloads, four pairs of ionizing radiation detectors, RadFETs, were deployed on the *O/OREOS* nanosat, a pair each on the exterior and the interior of each payload, SESLO and SEVO [28]. At 91 days, the exterior and interior SEVO detectors had measured total cumulative doses of ~2.8 and ~0.7 Gy, respectively [28]; the interior SESLO detectors measured ~0.5 Gy; this was consistent with the SESLO detectors being slightly more shielded than those of SEVO. To probe the effects of chronic exposure to the enhanced ionizing radiation environment of space in the high-inclination orbit used, two *B. subtilis* test strains were employed: wild-type strain 168 and ionizing radiation-sensitive strain WN1087, which is deficient in NHEJ-mediated DNA double-strand break repair [29]. No significant difference in the germination or growth of either strain was detected when comparing results for each from 14 and 91 days of spaceflight. This was not surprising, as the radiation dose inside the SESLO payload at 91 days, 0.5 Gy, was far below the dose required to cause 1 log of inactivation of even the radiation-sensitive mutant, calculated at ~100–200 Gy [13,29].

In contrast to the 14- and 91-day results, at 181 days it was observed that spore germination did not proceed normally and no reliable data were obtained. This was apparently not an effect of spaceflight per se, as both the FL and GC samples exhibited the same problem. We suspect that the multi-day thermal excursions from Days 102–116 and again from Days 162–176, peaking at 46.5 °C for the SESLO payload, could have been the cause of this failure. It appeared that the SESLO hardware (plumbing, optical system, telemetry) operated normally at 181 days, which would point to heat damage occurring either to the spores themselves or to some component(s) of the liquid growth medium. The spores were stored in the SESLO optical chambers as dried films, and the inactivation kinetics of dry spores at different temperatures have been well-characterized [30]. Based on the data presented in [30], we calculated that by heating to 44 °C, as occurred in the matched GC payload, it would take ~70 days for *B. subtilis* spores to lose 1 log of viability (i.e., the Decimal Reduction Time [31]); we estimate that the FL spores were between 41 and 44 °C for ~24 days in aggregate. We also ran the same calculation for scenarios of 50 °C and 60 °C, which yielded Decimal Reduction Times of ~35 days and ~14 days, respectively. Therefore, it seems unlikely that an aggregate 24-day thermal excursion to ≥ 44 °C resulted in such dramatic loss of spore viability. By process of elimination, one possibility is that some component(s) of the liquid growth medium (modified RPMI 1640 containing aB) may have been altered or destroyed by the prolonged period of heating. This is reasonable on the surface, as RPMI 1640 is a medium originally formulated for mammalian cell culture [32] and contains numerous amino acids and vitamins, some of which are heat labile (e.g., pyridoxine, folic acid). However, the *B. subtilis* strains used in the SESLO experiment are prototrophic for all growth factors except tryptophan, which is not itself heat labile, rendering this possibility less tenable. 

Alternatively, the excessive heating experienced by SESLO may have led to a biocompatibility issue with one or more of the components of the SESLO hardware, due for example to outgassing or leaching of harmful organic compounds; just such an effect appears to have contributed to the results recently reported for *E. coli* in the *EcAMSat* flight experiment [18]. The electronic printed circuit boards that support the LEDs, detectors, thermal control, operation of valves and pump, and thermal and radiation sensors are located within the same hermetically sealed payload volume as the microfluidic system, as are all of the aforementioned electrical and electromechanical components. For the SESLO experiment, the expulsion of air from the microfluidic wells that was necessary to initiate microbial growth required a pathway, via a hydrophobic porous membrane, from the microwells into the rest of the payload volume. Unfortunately, this pathway could also have allowed volatiles that might be evolved from the printed circuit board coating (Dow Corning 1-2577, a silicone resin) or electronic components to reach the spores. To minimize such effects, the printed circuit boards were off-gassed for ~14 days at 55 °C under vacuum prior to their integration with the rest of the payload; the payload also included activated carbon immobilized on a cloth carrier to absorb organic volatiles. Biocompatibility testing including pieces of the coated circuit board in proximity to dormant spores did not reveal adverse effect when spores were subsequently grown. However, it should be noted that the extensive pre-flight biocompatibility tests, to be reported elsewhere, were performed at nominal ambient temperature (~23 °C) and would not have uncovered such an off-nominal heating event. Regardless of the root cause, it is clear that the thermal-control systems of future nanosats will need to be engineered to tolerate a greater range of thermal stresses, particularly if being deployed to high-inclination/polar orbits in which multi-day periods with minimal eclipse are anticipated. 

## Supplementary Materials

(Archived Data) The complete set of SESLO data are available online at the NASA Ames Life Science Data Archive (LSDA) (https://www.nasa.gov/ames/research/space-biosciences/alsda) under accession number ARC00XX220.

## Appendix A

Optical transmittance through each 2.8 mm (diameter), 12 mm (length) well was monitored with using 3-color LED illumination (470, 525, and 615 nm) at one end of each well and an intensity-to-frequency detector at the other end (the output frequency is linearly proportional to input intensity). The wavelengths were chosen such that one (615 nm) is sensitive to the disappearance of the oxidized (blue) form of aB, and a second (525 nm) is sensitive mainly to the appearance of the reduced (pink) form of aB, with minor sensitivity to the blue form. The third wavelength (470 nm) has a lesser but finite sensitivity to the pink form of aB as well but no sensitivity to the blue form. All three wavelengths also are affected by optical density (light scattering) related to cell density. The reduction of the oxidized form of aB to its pink form is due to electron-transfer metabolites, including FADH (the semiquinone form of flavin adenine dinucleotide), NADH (the reduced form of nicotinamide adenine dinucleotide), and some of the more reducing cytochromes, all of which are general indicators of cellular metabolic activity.

Raw output frequency data was imported into Microsoft Excel and converted to Absorbance units as a function of time, *A*(*t*), using the following formula for each LED wavelength:

*A*(*t*) = [*Ao* + log (*fo*/*f*(*t*))]/*pl*,

where *Ao* is the laboratory-measured starting (unreacted) absorbance of the alamarBlue-containing growth medium at the wavelength of interest, including the optical density of the starting *B. subtilis* cells; *fo* is the frequency output of the optical sensor at time *t* = 0; *f(t)* is the frequency from the optical sensor at time *t*; *pl* = 1.2 is the optical pathlength of the Bioblock wells relative to a standard 1-cm optical pathlength. The complete sets of both raw and processed data are presented in the corresponding Tables in the LSDA.
